# Fisetin reduces the senescent tubular epithelial cell burden and also inhibits proliferative fibroblasts in murine lupus nephritis

**DOI:** 10.3389/fimmu.2022.960601

**Published:** 2022-11-17

**Authors:** Shogo Ijima, Yuki Saito, Kentaro Nagaoka, Sena Yamamoto, Tsukasa Sato, Norihiro Miura, Taiki Iwamoto, Maki Miyajima, Takako S. Chikenji

**Affiliations:** ^1^ Department of Oral Surgery, Sapporo Medical University School of Medicine, Sapporo, Japan; ^2^ Department of Anatomy, Sapporo Medical University School of Medicine, Sapporo, Japan; ^3^ Graduate School of Health Sciences, Hokkaido University, Sapporo, Japan

**Keywords:** systemic lupus erythematosus (SLE), lupus nephritis (LN), senescence, transforming growth factor β (TGF-β), p15^INK4B^, senolytic agent, fisetin

## Abstract

Systemic lupus erythematosus (SLE) is a chronic autoimmune inflammatory disease characterized by the involvement of multiple organs. Lupus nephritis (LN) is a major risk factor for overall morbidity and mortality in SLE patients. Hence, designing effective drugs is pivotal for treating individuals with LN. Fisetin plays a senolytic role by specifically eliminating senescent cells, inhibiting cell proliferation, and exerting anti-inflammatory, anti-oxidant, and anti-tumorigenic effects. However, limited research has been conducted on the utility and therapeutic mechanisms of fisetin in chronic inflammation. Similarly, whether the effects of fisetin depend on cell type remains unclear. In this study, we found that LN-prone MRL/lpr mice demonstrated accumulation of Ki-67-positive myofibroblasts and p15^INK4B^-positive senescent tubular epithelial cells (TECs) that highly expressed transforming growth factor β (TGF-β). TGF-β stimulation induced senescence of NRK-52E renal TECs and proliferation of NRK-49F renal fibroblasts, suggesting that TGF-β promotes senescence and proliferation in a cell type-dependent manner, which is inhibited by fisetin treatment *in vitro*. Furthermore, fisetin treatment *in vivo* reduced the number of senescent TECs and myofibroblasts, which attenuated kidney fibrosis, reduced senescence-associated secretory phenotype (SASP) expression, and increased TEC proliferation. These data suggest that the effects of fisetin vary depending on the cell type and may have therapeutic effects in complex and diverse LN pathologies.

## Introduction

Systemic lupus erythematosus (SLE) is an autoimmune disease characterized by the production of autoantibodies that affect multiple organs, including the heart, brain, skin, lungs, and kidneys. The most common and severe complication of SLE is lupus nephritis (LN), which is a major contributor to morbidity and mortality (1, 2). The pathogenesis of LN involves various factors, but its specific pathomechanisms remain unknown. Immunosuppressive agents and corticosteroids are standard treatments for patients with SLE. However, the long-term administration of these treatments is associated with numerous side effects, including osteoporosis, hypertension, diabetes, and infection (3). Therefore, a precise understanding of LN pathogenesis is essential for the development of new therapeutic targets.

Cellular senescence is characterized by irreversible cell cycle arrest, which can be triggered by many different factors including DNA damage, telomere dysfunction, oncogene activation, and organelle stress. The main physiological purpose of cellular senescence is to prevent the proliferation of damaged cells and trigger tissue repair through the secretion of various proteins—a phenotype termed as senescence-associated secretory phenotype (SASP) (4). However, aging or persistent damage causes the accumulation of senescent cells and impaired cell removal by the immune system, which can lead to the accumulation of chronic senescent cells and the promotion of fibrotic pathologies (5, 6).

Transforming growth factor β (TGF-β) is associated with several pathological processes, including renal fibrosis, promotion of myofibroblast differentiation, and accumulation of proteins and other components in the extracellular matrix (ECM) (7–9). In human glomerular diseases, increased TGF-β expression levels are observed in progressive glomerular diseases, and fibrotic areas are strongly correlated with TGF-β1 expression in biopsy specimens (10). TGF-β can also trigger the cellular senescence response, and TGF-β-mediated accumulation of senescent cells is implicated in idiopathic pulmonary fibrosis (6). TGF-β induces cyclin-dependent kinase inhibitor p15^INK4B^ and suppresses cell proliferation through a G1-phase cell cycle arrest. TGF-β induces or accelerates senescence and senescence-associated features in various cell types (11, 12). Although TGF-β induces cellular senescence in multiple cell types, it also induces proliferation in several types of mesenchymal cells. The stimulation of smooth muscle cells by TGF-β induces the expression of platelet-derived growth factor (PDGF) (13). In addition, cellular proliferation of human kidney cortical fibroblasts was induced *in vitro* by TGF-β treatment *via* the induction of basic fibroblast growth factor (FGF-2) (14). TGF-β1 promotes tubular and glomerular cell epithelial–mesenchymal transition (EMT), and the stimulated myofibroblasts produce excessive ECM and promote its deposition in the glomeruli and tubulointerstitium (15). TGF-β has different effects among different cell types, which may result in complex pathologies in chronic inflammation, including LN.

Senolytics—a class of drugs that selectively induce apoptosis in senescent cells—have attracted considerable attention as a novel therapeutic strategy against multiple chronic inflammatory diseases (16). Fisetin (3,3′,4′,7-tetrahydroxyflavone) is a senolytic that is a natural flavonoid found in various fruits and vegetables (17). It eliminates senescent cells by inducing apoptosis, thereby reducing chronic inflammation and fibrosis (16–18). Although fisetin functions as a senolytic, it also exerts anti-tumorigenic, antioxidant, anti-inflammatory, and anti-apoptotic effects (19–22). Furthermore, fisetin inhibits cell proliferation by upregulating p53 and p21, which are known senescence markers (23, 24). The effects of fisetin, such as senolysis and induction of cell senescence, appear to depend on the target cell type. Thus, several multifunctional effects of fisetin may be involved in treating the complex pathology of LN.

In this study, we hypothesized that TGF-β-related cellular senescence or proliferation occurs in LN and that the effects of fisetin vary according to the target cell type. We investigated the localization of TGF-β- and p15^INK4B^-positive cells in the LN of lupus-prone MRL/lpr mice. We found that p15^INK4B^-positive tubular epithelial cells (TECs) showed elevated TGF-β expression. Furthermore, the number of proliferative smooth muscle actin (SMA)-α-positive interstitial fibroblasts increased in MRL/lpr mice. *In vitro* experiments revealed that TGF-β stimulation triggered senescence in renal TECs, but promoted the proliferation of renal fibroblasts. Thus, fisetin treatment eliminates senescent TECs through its action as a senolytic and limits the proliferation of fibroblasts both *in vivo* and *in vitro*.

## Materials and methods

### Mice

Animal experiments were performed in accordance with the Guide for the Care and Use of Laboratory Animals and approved by the local review boards and authorities. Female MRL/lpr mice were used as SLE mouse models, and haplotype-matched female MRL/MpJ mice were used as phenotypic controls (Sankyo Lab Service). The mice were housed under specific pathogen-free (SPF) conditions in filter-top cages in rooms with constant temperature and humidity under a 12-h light/12-h dark cycle.

### Proteinuria

Proteinuria was assessed and scored semi-quantitatively using Albustix test strips (Siemens Healthineers). The scores were as follows: grade 0, 0 mg/dL; grade ±, <30 mg/dL; grade 1+, ≥30 mg/dL; grade 2+, ≥100 mg/dL; grade 3+, ≥300 mg/mL; and grade 4+, ≥1000 mg/dL.

### Cell culture and cell proliferation assays

NRK-52E rat renal proximal TECs (JCRB Cell bank: IFO50480) and NRK-49F rat renal fibroblasts (JCRB Cell bank: JCRB9067) were cultured in Dulbecco’s modified Eagle’s medium (DMEM) with non-essential amino acids containing 10% fetal bovine serum (FBS) or 5% FBS, respectively. For TGF-β stimulation, the cells were either untreated or treated with various concentrations (1, 5, or 10 ng/mL) of recombinant human TGF-β1 (240-b; R&D Systems) for two days. For fisetin treatment, the cells were untreated or treated with various concentrations (5, 10, or 20 µM) of fisetin dissolved in DMSO. Fisetin was purchased from Selleck (S2298). Cell proliferation was assessed using a Cell Counting Kit-8 (CK04; Dojindo).

### *In vivo* fisetin treatment

Eighteen-week-old MRL/lpr (n = 24) and MRL/MpJ mice (n = 24) were randomized for pharmacological treatment analysis, as previously described (25). Mice were orally administered 100 mg/kg fisetin (Tokyo Chemical Industry) (MRL/MpJ: n = 12 and MRL/lpr: n = 12) or vehicle (20% PEG 400) (MRL/MpJ: n = 12 and MRL/lpr: n = 12) five days a week for four weeks.

### Histology, immunohistochemistry, and immunofluorescence

Tissue samples were collected, fixed overnight in 4% paraformaldehyde (PFA) at 4°C, and embedded in paraffin. Paraffin-embedded tissues were sectioned (3-μm thickness) and stained with periodic acid-Schiff (PAS) for histological analysis. For immunohistochemical staining, the paraffin-embedded sections were deparaffinized and rehydrated for immunostaining. Antigen retrieval was performed in a microwave oven (95–98 °C for 10 min) using a citrate buffer (10 mM sodium citrate, pH 6.0). After cooling, the slides were washed twice with deionized water and once with 1X Tris-buffered saline with Tween-20 (TBST) for 5 min every time. The sections were blocked with 1% bovine serum albumin (BSA) in TBST for 15 min at room temperature (RT) and then incubated with primary antibodies overnight at 4 °C or for 1 h at RT. After washing thrice with TBST for 5 min each time, the sections were incubated with SignalStain Boost IHC Detection Reagent (HRP, Rabbit #8114; Cell Signaling Technology) for 30 min at RT in the dark. The sections were then washed in TBST thrice for 5 min each time and treated with TSA Plus Working Solution (Fluorescein, Cyanine 3, and Cyanine 5; AKOYA BIOSCIENCES) for 10 min at RT in the dark. For multiplex staining, stripping was performed in a microwave oven (95–98 °C for 10 min) using citrate buffer. After cooling, staining with different tyramide fluorescent labels was performed as described above. The following antibodies were used: anti-Col1 (Abcam, ab34710, rabbit polyclonal, 1:300), anti–TGF-β1 (ProteinTech, 21898-1-AP, rabbit polyclonal, 1:400), anti–α-SMA (Cell Signaling Technology, D4K9N, 1:500), anti-p15^INK4b^ (Abcam, ab53034, rabbit polyclonal, 1:500), anti-CD4 (Cell Signaling Technology, D7D2Z, 1:200), anti-CD8a (Cell Signaling Technology, D4W2Z, 1:800), anti-F4/80 (Cell Signaling Technology, D2S9R, 1:500), and anti-Sox9 (Cell Signaling Technology, D8G8H, 1:800). Nuclei were stained using 4’,6-diamidino-2-phenylindole (DAPI) (Dojindo). Sections were observed under a fluorescence microscope (Axio Observer 7; Zeiss). The fluorescence intensities of p15^INK4B^ and TGF-β1 were analyzed using the image analysis function of ZEN (Zeiss). Photographs (×20 magnification) of randomly selected tubules and glomeruli were analyzed, and the average intensities of p15^INK4B^ and TGF-β1 per area were obtained. For tubule analysis, nine tubules from each of the three fields per mouse were randomly selected and evaluated. For glomerular analysis, 2–3 glomeruli in each of the three fields per mouse were randomly selected and evaluated. The number of α-SMA-, Ki-67-, CD4-, CD8-, F4/80-, and Sox9-positive cells was counted manually under blinded conditions. Cultured cells were fixed with 4% paraformaldehyde for 15 min at RT, incubated in 0.01 M phosphate-buffered saline (PBS) containing 0.3% Triton (PBS-T), and then treated with 2% BSA for 60 min at RT. After washing with PBS-T, the cells were incubated first with the primary antibodies anti-p15^INK4B^ (Abcam, polyclonal, 1:500), anti-γH2AX (Cell Signaling Technology, 20E3, 1:400), anti–phospho-mTOR(Ser2448) (Sigma-Aldrich, 1C22, 1:100), and anti-Smad2/3 (Cell Signaling Technology, D7G7, 1:400) and then with the secondary antibody Alexa Fluor Plus 555 (Invitrogen). The nuclei were stained with DAPI (Dojindo). The cells were observed under a fluorescence microscope (ZEISS) and analyzed using ImageJ software (National Institutes of Health). The percentage of p15^INK4B^-positive cells was calculated by dividing the number of p15^INK4B^-positive cells by the total number of DAPI-positive cells. The number of foci of γH2AX (histone H2AX phosphorylation, a marker of DNA damage–related senescence (26)) was counted using ImageJ software and normalized against the total cell number in each image. To count the γH2AX foci, the TIFF images were imported into ImageJ and converted into 8-bit images. The “Binary and Threshold” functions of ImageJ were used to detect γH2AX foci, which were counted using the “Analyze Particles” function. The counted γH2AX foci number was divided by the number of DAPI-stained cells. To analyze the intensity corresponding to phospho-mTOR and Smad2/3 proteins, the cells were randomly selected using the “Regions of interest (ROI)” function and analyzed using the “Measure” function of ImageJ. For SPiDER-β-gal staining, the cells were washed twice with PBS, fixed in 4% PFA at RT for 5 min, and washed twice again with PBS. The sections were incubated in 20 µM SPiDER-β-gal (Dojindo) in McIlvaine buffer (pH 6.0) for 60 min at 37°C. After washing the tissue sections, the nuclei were stained with DAPI.

### Histopathological evaluation of the kidney

PAS-stained paraffin sections were used for morphological evaluation, and both glomerular inflammation (hypercellularity, mesangial matrix expansion, and crescent formation) and interstitial inflammation were graded on a scale of 0–3 as previously described by Pérez de Lema et al. (27). Hypercellularity, mesangial matrix expansion, and interstitial inflammation were classified as follows: grade 0, absence; grade 1, mild; grade 2, moderate; and grade 3, severe. Crescent formation was defined as follows: grade 0, <10%; grade 1, 10–25%; grade 2, 25–40%; and grade 3, >40%.

### RNA extraction and quantitative real-time PCR

Total RNA was isolated from cultured cells and tissues using TRI Reagent (Molecular Research Center) and was reverse transcribed into cDNA using the iScript Advanced cDNA Synthesis Kit (Bio-Rad). Quantitative PCR was performed using the SsoAdvanced Universal SYBR Green Supermix (Bio-Rad) in a CFX Connect Real-Time PCR Detection System (Bio-Rad) under the following cycling conditions: 95°C for 30 s, followed by 40 cycles of amplification (95°C for 10 s and 60°C for 30 s). The primer sequences used for the PCR are listed in **Supplementary Table 1**. The samples were compared using the ΔΔCt method.

### ELISA

Blood samples were collected from the mice after fisetin treatment through cardiac puncture under anesthesia at the time of euthanization. The concentration of anti-dsDNA antibodies was measured using an ELISA kit (AKRDD-061, Fujifilm Wako).

### Gene expression omnibus data

Gene expression data of patients with LN were obtained from the NCBI Gene Expression Omnibus (GEO) using the GEOquery R package (28). The accession number of the dataset is GSE200306. The dataset includes 10 control and 45 LN tubulointerstitial samples, 9 control glomeruli samples, and 34 LN glomeruli samples.

### Statistical analysis

Quantitative data are reported as means and medians with interquartile ranges (IQRs) and 1.5 times the IQR. Data were plotted using dot plots and box and violin plots using ggplot2, ggpubr, and gplots, which are plotting systems for R based on the Grammar of Graphics (The R Foundation for Statistical Computing). Normality was assessed using the Shapiro–Wilk test. The pairwise t-test or two-tailed Mann–Whitney U test was used for comparisons between the two groups. One-way analysis of variance (ANOVA) was conducted to assess the differences among three or more groups. *P*-values for multiple comparisons were adjusted using the Tukey’s method. Pearson’s correlation coefficient was used to assess the correlations. Statistical analyses were performed using EZR, a graphical user interface for R (29). Two-sided *p*-values less than 0.05 were considered statistically significant.

## Result

### Expression of TGF-β and p15^INK4B^ is elevated in TECs

**Figure 1:**
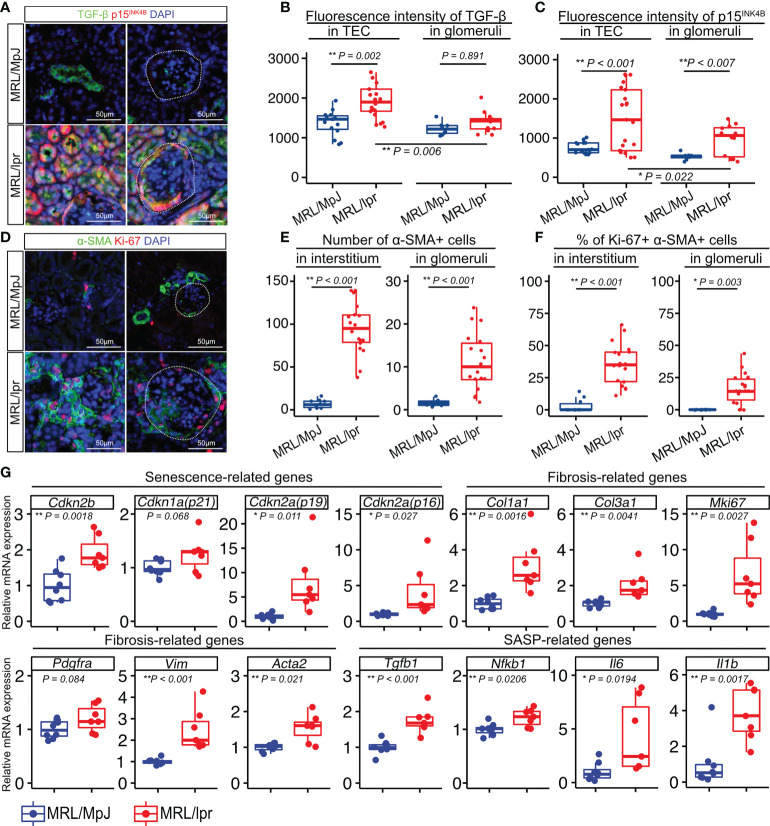
Numbers of senescent cells and proliferating cells are increased in the kidneys of MRL/lpr mice. **(A)** Representative images of immunohistochemical staining for TGF-β1 and p15^INK4B^ in kidney sections from MRL/lpr and MRL/MpJ (control) mice. **(B–C)** Quantification of TGF-β1 and p15^INK4B^ expression levels in tubular epithelial cells (TEC) and glomeruli measured using fluorescence intensity (arbitrary units, a.u.). **(D)** Representative images of immunohistochemical staining for α-SMA and Ki-67 in kidney sections from MRL/lpr and MRL/MpJ mice. **(E–F)** Quantification of the number of α-SMA-positive cells and the percentage of Ki-67- and α-SMA-positive cells in the interstitium and glomeruli. **(G)** Relative mRNA expression of senescence-related genes (*Cdkn2b, Cdkn1a, Cdkn2a(p19), Cdkn2a(p16))*, fibrosis-related genes *(Col1a1, Col3a1, Mki67, Pdgfra, Vim, Acta2)*, and SASP-related genes *(Tgfb1, Nfkb1, Il6, Il1b)* in the kidneys of MRL/lpr and MRL/MpJ (control) mice. Data are presented as medians with IQRs and 1.5 times the IQR and are displayed as dot plots and box-and-whisker plots. *P*-values were determined using one-way ANOVA adjusted by Tukey’s method or the two-tailed Student’s t-test. (*P < 0.05 and **P < 0.01). Scale bars represent 50 µm.

To investigate if TGF-β is associated with LN, we examined the cellular expression of TGF-β in the LNs of 18-week-old MRL/lpr mice and age-matched MRL/MpJ mice as controls. MRL/lpr mice have a mutation in the gene encoding Fas(lpr) and develop a syndrome resembling human SLE-induced LN (30, 31). First, we confirmed that these mice exhibited more severe proteinuria and higher histopathological scores for the glomeruli and tubulointerstitium than did the controls (**Supplementary Figures 1A–D**). To examine ECM deposition in the kidneys, type I collagen (Col1) was stained using immunohistochemistry. Col1 expression was increased in both glomeruli and tubulointerstitium of MRL/lpr mice (**Supplementary Figures 1E–G**), suggesting that these mice had increased fibrotic areas in the kidney. Next, we performed immunohistochemical analysis to identify the location of the TGF-β1- and p15^INK4B^-expressing cells. Compared with that in control mice, MRL/lpr mice showed higher TGF-β1 expression in TECs, but not in the glomeruli (**Figures 1A, B**). Using NCBI’s Gene Expression Omnibus (32), we also found that *TGFB1* expression was significantly increased in the tubulointerstitium of LN patients, but not in the glomeruli (**Supplementary Figure 2**). The expression of p15^INK4B^ in both TECs and glomeruli was higher in MRL/lpr mice than that in control mice (**Figures 1A, C**). In addition, MRL/lpr mice exhibited higher expression of TGF-β1 and p15^INK4B^ in the TECs than in the glomeruli (**Figures 1A–C**). To determine whether p15^INK4B^ is a senescence marker, we performed co-immunostaining using anti-p15^INK4B^, p16^INK4A^, and γH2AX antibodies. We found that 89% of the p15^INK4B^ cells expressed either p16^INK4A^, γH2AX, or both (**Supplementary Figure 3**). α-smooth muscle actin (α-SMA), which is expressed on myofibroblasts and mesangial cells in chronic glomerulonephritis (33), was present at higher levels in MRL/lpr mice than in control mice (**Figures 1D, E**). Furthermore, the percentages of Ki-67+ proliferating myofibroblasts and mesangial cells increased in MRL/lpr mice (**Figures 1D, F**). PCR analysis revealed that compared with that in control mice, MRL/lpr mice showed increased expression of senescence-related genes (*Cdkn1a*, *Cdkn2a* (p16 and p19), and *Cdkn2b*), SASP-related genes (*Il1b*, *Tgfb1*, *Il6*, and *Nfkb1*), and fibrosis-related genes (*Col1a1*, *Col3a1*, *Mki67, Pdgfra, Vim, and Acta2*) (**Figure 1G**). These results suggested that the kidneys of MRL/lpr mice were characterized with not just nephropathy, but with increased numbers of senescent TECs and proliferating α-SMA-positive myofibroblasts.

### TGF-β induces renal TEC senescence and promotes renal fibroblast proliferation

**Figure 2:**
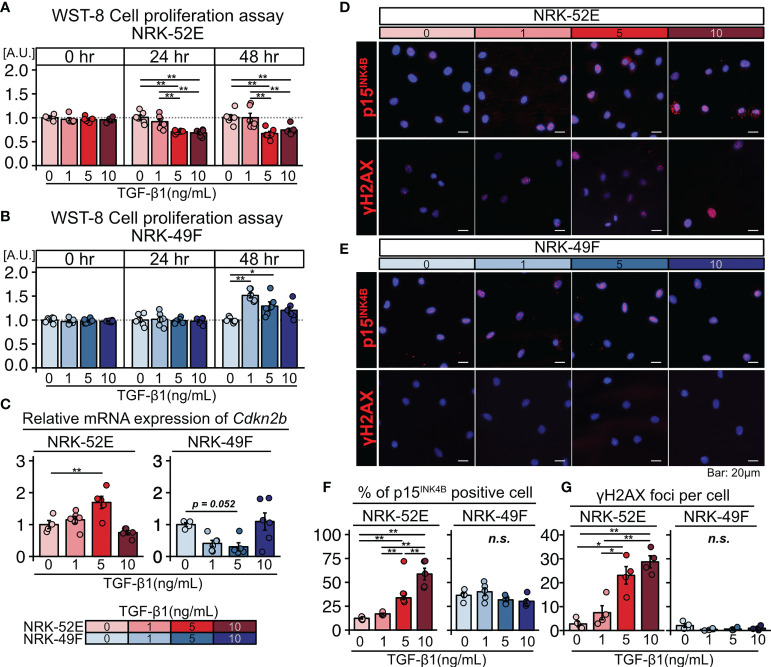
TGF-β induces senescence in renal tubular cells and the proliferation of renal fibroblasts. **(A, B)** WST-8 cell proliferation assay in NRK-52E normal renal proximal tubular cells **(A)** and NRK-49F normal renal fibroblasts **(B)** cultured with and without TGF-β1 stimulation (1, 5, or 10 ng/ml). **(C)** Relative mRNA expression of *Cdkn2b* in NRK-52E and NRK-49F cells with and without TGF-β1 stimulation (1, 5, or 10 ng/mL). **(D, E)** Representative images of p15^INK4b^ and γH2AX staining in NRK-52E and NRK-49F cells with and without TGF-β1 stimulation (1, 5, or 10 ng/mL). **(F, G)** Percentage of cells positive for p15^INK4B^ and the number of γH2AX foci. *P*-values were determined using one-way ANOVA adjusted by Tukey’s method (*P < 0.05 and **P < 0.01).

Based on the histological analysis described above, we hypothesized that TGF-β triggers senescence in renal TECs in an autocrine manner and proliferation of renal fibroblasts in a paracrine manner. To test this hypothesis, we cultured the normal renal proximal tubular cell line NRK-52E and normal renal fibroblast line NRK-49F with and without TGF-β1 stimulation (1, 5, or 10 ng/mL) for two days. The WST-8 assay revealed that proliferation decreased in NRK-52E cells (**Figure 2A**), but increased in NRK-49F cells following TGF-β1 stimulation (**Figure 2B**). Furthermore, NRK-52E cells showed increased *Cdkn2b* (p15^INK4B^) mRNA expression in response to TGF-β1 treatment, whereas NRK-49F cells did not (**Figure 2C**). The protein expression of p15^INK4B^ and γH2AX was also increased in NRK-52E cells following TGF-β1 stimulation, whereas that of NRK-49F was not (**Figures 2D–G**). These results suggest that TGF-β induces cellular senescence in renal TECs but promotes the proliferation of renal fibroblasts. They also suggest that the diverse functions of TGF-β may induce TEC senescence and interstitial fibrosis in LN.

### Fisetin treatment reduces SPiDER-β-gal expression in NRK-52E cells and inhibits TGF-β-induced activation of NRK-49F cells

**Figure 3:**
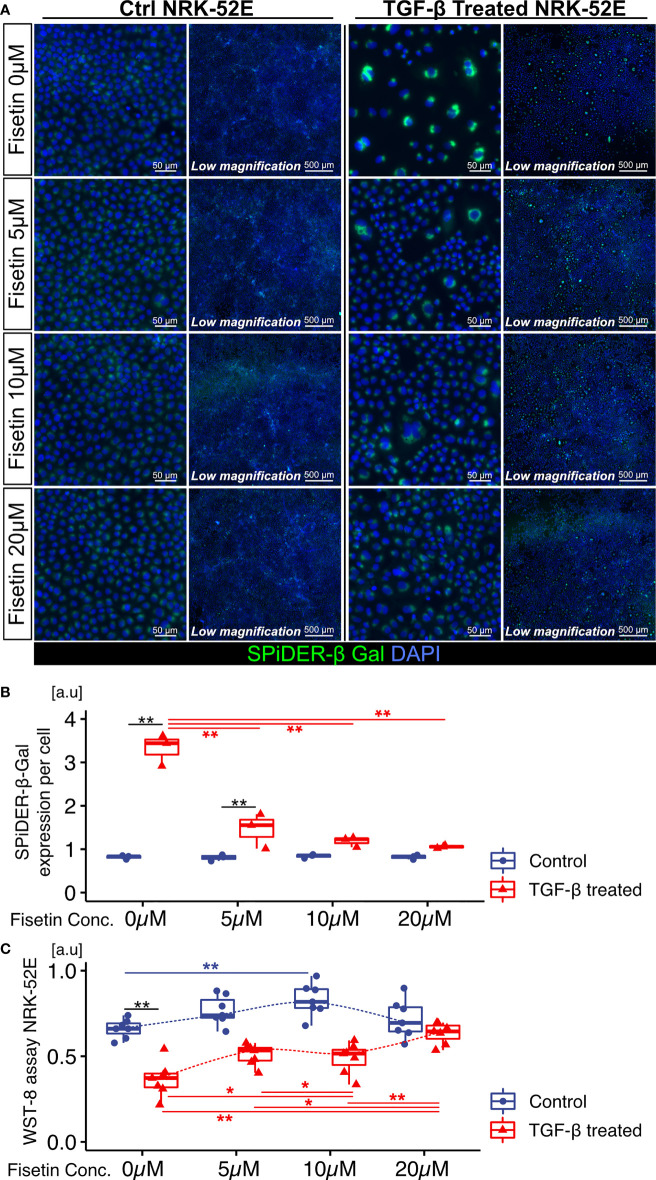
Fisetin treatment reduces the number of SPiDER-β-Gal–positive cells in TGF-β1–treated senescent renal tubular cells. **(A)** Representative images of SPiDER-β-Gal staining in TGF-β1–treated senescent NRK-52E cells with and without fisetin treatment (0, 5, 10, 20 µM). **(B)** Quantification of SPiDER-β-Gal expression levels in NRK-52E cells measured based on fluorescence intensity. **(C)** WST-8 cell proliferation assay in NRK-52E cells after TGF-β1 and fisetin treatment. *P*-values were determined using two-way ANOVA adjusted by Tukey’s method (*P < 0.05 and **P < 0.01).

**Figure 4:**
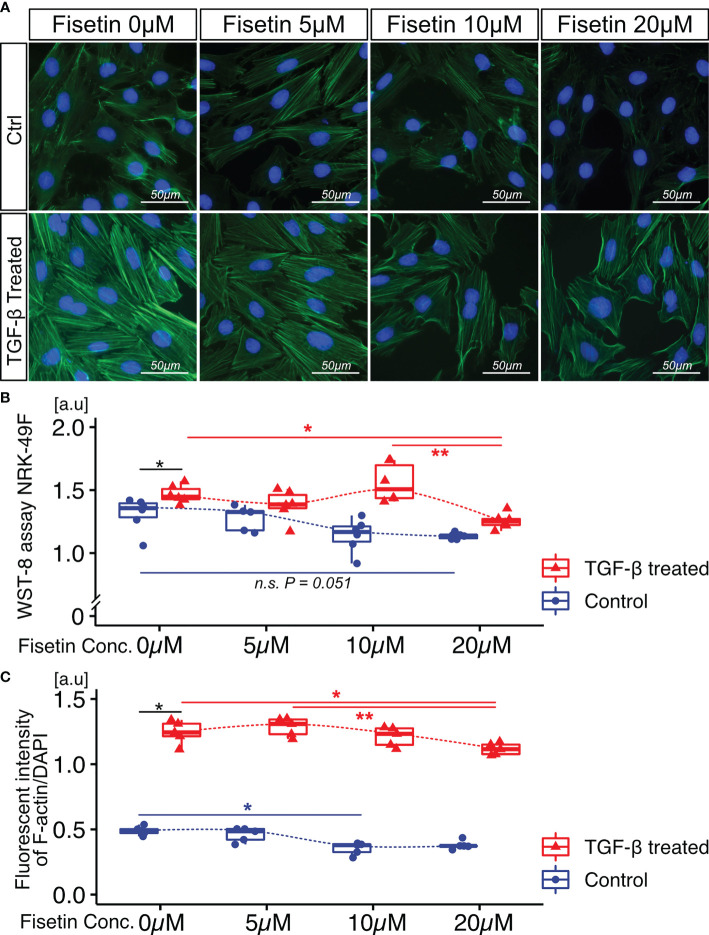
Fisetin treatment reduces cell proliferation and F-actin expression in TGF-β1–stimulated NRK-49F cells. **(A)** Representative images of F-actin staining in TGF-β1–stimulated NRK-49F cells with and without fisetin treatment (0, 5, 10, 20 µM). **(B)** WST-8 cell proliferation assay of NRK-49F cells after TGF-β1 and fisetin treatment. **(C)** Quantification of F-actin expression levels in NRK-49F cells measured using a fluorescence microplate reader. *P*-values were determined using two-way ANOVA adjusted by Tukey’s method (*P < 0.05 and **P < 0.01).

Next, we tested the effects of fisetin on TGF-β-induced senescence in NRK-52E cells. Fisetin has been shown to have a therapeutic effect on neuropsychiatric symptoms in MRL/lpr mice (25). Senescent NRK-52E cells with and without TGF-β induction were treated with serial concentrations of fisetin (0–20 µM) for 24 h. To detect senescence-associated β-galactosidase, we used SPiDER-β-Gal, which is a fluorescent probe that exhibits fluorescence activation upon reaction with β-galactosidase (34). TGF-β-treated cells showed increased SPiDER-β-Gal expression and decreased cell proliferation (**Figures 3A–C**). The doses of 5, 10, and 20 µM fisetin decreased SPiDER-β-Gal expression in TGF-β-treated senescent NRK-52E cells (**Figures 3A, B**). In the cell proliferation assay, control NRK-52E cells exhibited increased cell proliferation at a fisetin dose of 10 µM, and fisetin-treated senescent NRK-52E cells demonstrated increased cell proliferation in a dose-dependent manner (**Figures 3A, C**). Furthermore, TGF-β treatment increased proliferation and F-actin expression in NRK-49F cells (**Figures 4A–C**). A fisetin dose of 20 μM decreased the proliferation of TGF-β-treated NRK-49F cells and F-actin expression (**Figures 4A–C**). In addition, fisetin doses of 10 and 20 µM decreased phospho-mTOR expression, but did not inhibit the nuclear translocation of Smad2/3 (**Supplementary Figures 4A–D**). Based on these results, we hypothesized that fisetin decreases the number of senescent TECs and inhibits fibroblast proliferation in MRL/lpr kidneys.

### Fisetin treatment reduces the expression of p15^INK4B^ in TECs

**Figure 5:**
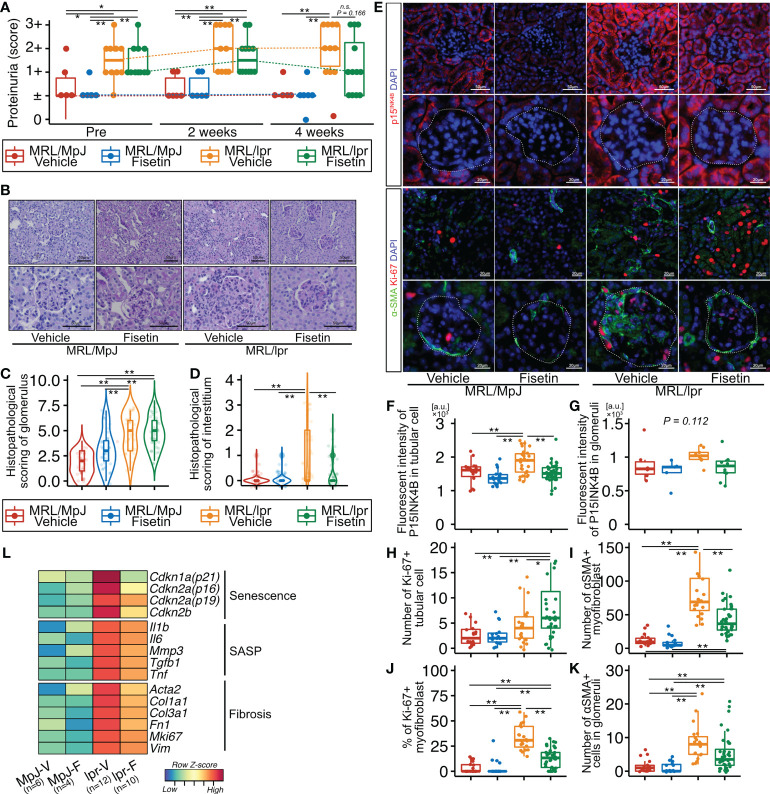
*In vivo* fisetin treatment reduces expression of p15^INK4B^ and SASP genes and inhibits α-SMA–positive myofibroblasts in MRL/lpr mice. **(A)** Semi-quantitative data of proteinuria after fisetin treatment. **(B–D)** Representative images of PAS-stained kidneys from vehicle- and fisetin-treated MRL/MpJ and MRL/lpr mice **(B)**, and quantification of the histopathological scores of the glomeruli and interstitium **(C**, **D)**. **(E)** Representative images of immunohistochemical staining for p15^INK4B^ in kidney sections from MRL/lpr and MRL/MpJ mice after fisetin treatment. **(F–H)** Quantification of fluorescence intensity of p15^INK4B^ expression in tubular cells and glomeruli **(F, G)** and the number of Ki-67-positive tubular cells **(H)**. **(I, J)** Quantification of the number of α-SMA–positive cells **(I)** and the percentage of Ki-67- and α-SMA-positive cells in the interstitium **(J)**. **(K)** Quantification of the number of α-SMA-positive cells in glomeruli. **(L)** Heatmap of differentially expressed senescence-, fibrosis-, and SASP-related genes. Higher expression is depicted in red, lower expression is depicted in blue, and equivalent expression is depicted in yellow. Data are presented as medians with IQRs and 1.5 times the IQR and are displayed as dot plots and box-and-whisker plots. *P*-values were determined using one-way ANOVA adjusted by Tukey’s method. (*P < 0.05 and **P < 0.01).

To examine the effect of fisetin *in vivo*, we orally administered fisetin (100 mg/kg) or 20% PEG400 (as a control) to MRL/lpr and MRL/MpJ mice for five days a week for four weeks. The proteinuria score was significantly higher in MRL/lpr mice than in MRL/MpJ mice before treatment, but this score did not differ significantly between MRL/lpr and MRL/MpJ mice after four weeks of fisetin treatment (**Figure 5A**). The concentration of dsDNA autoantibodies was significantly higher in vehicle-treated MRL/lpr mice than in vehicle-treated MRL/MpJ mice; however, there was no significant difference between vehicle-treated MRL/MpJ mice and fisetin-treated MRL/lpr mice (**Supplementary Figure 5**). Histopathological analysis showed that four-week fisetin treatment in MRL/lpr mice did not improve the histopathological scores of glomeruli, but did significantly decrease cell infiltration in the interstitium (**Figures 5B–D**). IHC analysis showed increased presence of CD4-, CD8-, and F4/80-positive cells in vehicle-treated MRL/lpr mice, and these cells were decreased on fisetin treatment in MRL/lpr mice (**Supplementary Figures 6A, B**). Fisetin also reduced the expression of p15^INK4B^ in TECs and increased the number of KI-67+ TECs in MRL/lpr mice (**Figures 5E–H**). In addition, fisetin inhibited the accumulation of αSMA+ myofibroblasts and decreased the number of KI-67+ αSMA+ myofibroblasts in the interstitium (**Figures 5E, I, and J**). The number of αSMA+ cells and p15^INK4B^ expression levels in the glomeruli were not affected by fisetin treatment in MRL/lpr mice (**Figures 5E, G, and K**). The number of cells positive for SOX9—a marker of renal stem/progenitor cells (35)—was higher in fisetin-treated MRL/lpr mice than in vehicle-treated MRL/lpr mice (**Supplementary Figures 7A, B**). PCR analysis showed that fisetin treatment decreased the expression of senescence-related genes (*Cdkn1a*, *Cdkn2a* (p16 and p19), and *Cdkn2b*), SASP-related genes (*Tnf*, *Mmp3*, *Il1b*, *Tgfb1*, and *Il6*), and fibrosis-related genes (*Col1a1*, *Col3a1*, *Fn1*, *Mki67*, *Acta2*, and *Vim*) (**Figure 5L**).

## Discussion

Cellular senescence is a stress-induced growth arrest that has been observed in multiple kidney diseases. Senescent TECs were present in 80% and 21% of the patients with and without kidney disease, respectively (36, 37). Furthermore, patients with IgA nephropathy demonstrated increased p21^Cip1^ and p16^INK4A^ protein expression confined to the tubules (36, 38). TEC senescence was also observed in the early phase after acute kidney injury in various mouse models (39). Patients with LN demonstrated increased renal p16^INK4A^ expression, which was associated with more severe fibrosis and greater CD8+ T-cell infiltration (40). Another study reported that p16^INK4A^ expression was increased in TECs (41). We demonstrated that p15^INK4B^-expressing TECs with high TGF-β expression accumulated in MRL/lpr mice—an animal model of LN. Although various senescence-related genes and proteins are involved in chronic inflammation in multiple organs, we focused on p15^INK4B^, which is upregulated during TGF-β-related cell senescence (42). p15^INK5B^, encoded by *CDKN2B*, is located near *CDKN2A*, which is another member of the INK4 family. p15^INK4B^ binds to CDK4 and CDK6, preventing their binding to cyclins, thereby inhibiting cell cycle progression (42). Another means by which TGF-β prevents cell proliferation is by inhibiting c-Myc expression. c-Myc is a key transcription factor involved in regulating cell growth, and it inhibits the expression of p15^INK4B^ and p21^Cip1^ in proliferating cells (43). Suppression of c-Myc expression by TGF-β limits c-Myc availability and suppresses p15^INK4B^ and p21^Cip1^ function (13, 44). In addition, the upregulation of p15^INK4B^ expression in response to TGF-β is mediated by Smad-induced transcriptional activation. FoxO proteins, a family of transcription factors that interact with Smads, upregulate CDKN2B expression (45). The interaction between Smads and Sp1 at the *CDKN2B* promoter also contributes to the induction of these genes in response to TGF-β (46). For the analysis of senescence markers in this study, we included p15^INK4B^, p16^INK4A^, and γH2AX. Although terminally differentiated cells such as podocytes may also express cell cycle arrest markers, a recent study showed that p16^INK4A^ expression levels in podocytes were positively correlated with age and levels of other SASP factors in mice (47). We found that 89% of p15^INK4B^-positive cells exhibited co-expression of the senescence markers p16^INK4A^ and γH2AX. An increased number of senescent cells may be a pathological change characteristic of LN, and terminally differentiated cells may not be eliminated. Further research is needed to investigate the specific markers that identify senescent cells.

This study demonstrated that MRL/lpr mice exhibited an increased number of p15^INK4B^-expressing TECs and Ki-67-expressing myofibroblasts. Although TGF-β induces antiproliferative effects in multiple cell types, such as epithelial, endothelial, and neuronal cells, it also stimulates cell proliferation in other mesenchymal cell types (48, 49). Strutz et al. demonstrated that TGF-β1 stimulation induces the proliferation of human renal fibroblasts *via* the induction of basic fibroblast growth factor (14). Battegay et al. demonstrated that stimulation of TGF-β in smooth muscle cells induced the expression of PDGF and the autocrine effect of PDGF-induced cell proliferation (13). TGF-β-stimulated proliferation of smooth muscle cells only occurs at low TGF-β concentrations, whereas higher concentrations limit cell proliferation (13). The molecular mechanism underlying this observation is still unclear, but cell type and TGF-β expression level may affect both cell senescence and proliferation in SLE-induced LN.

Fisetin—a flavonoid found in many fruits and vegetables (22, 50)—has a variety of functional properties such as reducing inflammation and neutralizing reactive oxygen species (51–53). It also exerts antitumor activities. For instance, by inhibiting Ki-67 expression, it blocks the PI3K/AKT/mTOR pathway; by acting as a topoisomerase inhibitor it decreases cell proliferation (54–56). Inhibition of PI3K/mTOR suppresses activated myofibroblasts and possibly induces apoptosis (57). Fisetin has also recently been found to have senolytic effects, selectively killing senescent cells by inhibiting the anti-apoptotic pathway of senescent cells, which includes the PI3K/AKT/mTOR pathway (58, 59). We found that fisetin treatment inhibited phospho-mTOR expression in TGF-β-treated senescent NRK-52E cells *in vitro* but did not inhibit Smad2/3 nuclear translocation. Fisetin may inhibit the PI3K/AKT/mTOR pathway in senescent TECs in a Smad-independent manner, resulting in the attenuation of kidney fibrosis. Fisetin may also exert a therapeutic effect by suppressing renal proinflammatory cell infiltration or the production of cytokines such as T-cell-derived TNFα and macrophage-derived TGF-β, both of which can induce cell senescence (60). We found that CD4-, CD8-, and F4/80-positive cells were increased in vehicle-treated MRL/lpr mice, and these cell numbers were decreased on fisetin treatment. Yet, this still does not indicate whether fisetin treatment reduces senescent cells directly or indirectly; in fact, both are possible.

The inhibitory effect of fisetin on cell proliferation depends on the cell type and dose. Fisetin inhibited the proliferation of SGC7901 cells (a human gastric cancer cell line) at a dose of 5–20 µM (61). Fisetin also promotes the proliferation of other cell types, such as the human keratinocyte cell line (HaCaT) and human foreskin fibroblasts (Hs68) at a dose of 5–10 µM (62, 63). Although the detailed underlying mechanisms remain unknown, fisetin activates the expression of telomerase reverse transcriptase (TERT), insulin-like growth factor (IGF)-1, and keratinocyte growth factor (KGF) (63), all of which promote epithelial cell proliferation (64). Treatment with 10 µM of fisetin may affect the proliferation of NRK-52E control cells.

Interestingly, our *in vitro* and *in vivo* experiments showed that fisetin treatment promoted the proliferation of non-senescent TECs. The adult kidney is characterized by minimal proliferation of TECs (65, 66). However, TECs rapidly enter the cell cycle after tissue damage, and their proliferative capacity may contribute to the replacement of dead cells. This contributes to kidney regeneration (65–67) *via* dedifferentiation of proximal tubule epithelial cells, which act as adult renal stem/progenitor cells (66, 67), or *via* the proliferation of new epithelial cells that arise from the self-duplication of surviving cells rather than from a specialized progenitor cell population (65). Our *in vitro* experiment showed a decrease in the number of NRK-52E cells located near SPiDER-β-Gal-positive senescent NRK-52E cells, and non-senescent NRK-52E cell numbers increased after the elimination of senescent NRK-52E cells by fisetin treatment. Cells in the vicinity of senescent cells undergo cellular senescence *via* the SASP and experience cell cycle arrest (68, 69). Fisetin treatment may activate renal stem/progenitor cells and induce tubular cell proliferation either directly or by eliminating senescent cells. In this study, we found that the number of SOX9-positive cells increased after fisetin treatment in the MRL/lpr mice. Previous studies have shown that Sox9 activation occurred after kidney injury and promoted regeneration (35, 70–72). Although the number of Sox9-positive renal stem/progenitor cells was relatively low, our results showed that fisetin treatment could potentially increase their number and contribute to kidney regeneration, which may reduce proteinuria in LN mice. Further studies are needed to confirm these findings and clarify the underlying mechanisms. Fisetin also increases the expression of Ccnd2, Cdk6, Ccne1, and Cdk1, which are required for the cell cycle G1/S transition (73). Furthermore, fisetin inhibits ROS production and caspase 9 expression (51, 73), which may promote TEC survival after tissue damage. In the future, examination of the mechanism by which fisetin treatment restores TECs in the context of LN would need to be explored. In the present study, in an LN model, fisetin reduced the accumulation of senescent tubular epithelial cells and inhibited fibroblast proliferation and, by extension, renal fibrosis. This improved renal function possibly by reducing proteinuria. Previous studies have shown that improving renal fibrosis reduces proteinuria (74, 75). Conversely, improving proteinuria prevented renal fibrosis (76, 77), suggesting that proteinuria and renal fibrosis play reciprocal roles. Taken together, the clearance of senescent tubular epithelial cells and inhibition of renal fibrosis may improve protein reabsorption in the LN model, although it remains unclear why senescent glomerular cells were not removed. Further research is needed to investigate why the pathological changes improved in the LN model.

We demonstrated that fisetin treatment reduced the number of senescent TECs and proliferating myofibroblasts, but could not identify a significant therapeutic effect on the glomeruli. Several studies have shown that 8–16 weeks of fisetin treatment attenuated diabetic nephropathy in mouse models of streptozotocin- or a high-fat diet-induced diabetic nephropathy (78, 79). In our study, fisetin was administered for four weeks. Although previous studies have shown that a four-week fisetin treatment decreased the number of senescent cells (25, 80), this treatment duration may be insufficient to attenuate LN. Further research is needed to determine whether long-term fisetin treatment can attenuate nephropathy in SLE.

In conclusion, we demonstrated that p15^INK4B^-positive TECs and Ki-67-positive myofibroblasts accumulated in LN-prone MRL/lpr mice. The p15^INK4B^-positive TECs in LN exhibited high TGF-β expression. TGF-β stimulation induced senescence of NRK-52E renal TECs and proliferation of NRK-49F renal fibroblasts, suggesting that TGF-β promotes cell senescence and proliferation in a cell type-dependent manner as well as providing novel insights into the complex pathology of LN. Furthermore, *in vivo* fisetin treatment reduced the number of senescent TECs and myofibroblasts, thus attenuating kidney fibrosis, decreasing SASP expression, and increasing TEC proliferation.

## Data availability statement

The raw data supporting the conclusions of this article will be made available by the authors, without undue reservation.

## Ethics statement

The animal study was reviewed and approved by The Committee of the Animal Experimentation Center of the Sapporo Medical University School of Medicine.

## Author contributions

SI, collection and assembly of data and manuscript writing. YS, conception and design, collection and assembly of data, and manuscript writing. KN, collection and assembly of data. SY, TS, NM, TI, and MM, data collection. TC, conception and design, collection and assembly of data, manuscript writing, and final approval of manuscript. All authors contributed to the article and approved the submitted version.

## Funding

This study was supported by JSPS KAKENHI (Grant Numbers 21H03049, 21H03293, and 18K16668), Sapporo medical university research grant.

## Acknowledgments

The authors thank Yumiko Takagi and Naoko Sai for their technical support. We would like to thank Editage (www.editage.com) and Zenis Co. Ltd. for English language editing.

## Conflict of interest

The authors declare that the research was conducted in the absence of any commercial or financial relationships that could be construed as a potential conflict of interest.

## Publisher’s note

All claims expressed in this article are solely those of the authors and do not necessarily represent those of their affiliated organizations, or those of the publisher, the editors and the reviewers. Any product that may be evaluated in this article, or claim that may be made by its manufacturer, is not guaranteed or endorsed by the publisher.
