# Supplementary material for: Fisetin reduces the senescent tubular epithelial cell burden and also inhibits proliferative fibroblasts in murine lupus nephritis
Source: Front Immunol. 2022 Nov 17;13:960601. doi: 10.3389/fimmu.2022.960601 (PMC9714549; doi:10.3389/fimmu.2022.960601)
Supplement: Supplementary file 1 [file DataSheet_1.pdf]

Supplementary File for

Fisetin reduces the senescent tubular epithelial cell burden and also inhibits proliferative fibroblasts in murine lupus nephritis

Shogo Ijima[1], Yuki Saito[2*], Kentaro Nagaoka[2], Sena Yamamoto[3], Tsukasa Sato[3], Norihiro Miura[3], Taiki Iwamoto[2], Maki Miyajima[3], and Takako S. Chikenji[2,3*]

[1]Department of Oral Surgery, Sapporo Medical University School of Medicine, Sapporo, Japan

[2]Department of Anatomy, Sapporo Medical University School of Medicine, Sapporo, Japan

[3]Department of Health Sciences, School of Medicine, Hokkaido University, Sapporo, Japan

*** Correspondence:**
Takako S. Chikenji, PhD

Address: North12 West5, Kitaku, Sapporo, 060-0812, Japan

Telephone: 011-706-3382 FAX: 011-706-3382

Email: chikenji@pop.med.hokudai.ac.jp

Yuki Saito, Ph.D.

Address: South1 West17, chuoku, Sapporo, 060-8556, Japan

Telephone: 011-611- 2111

Email: yuki.saito@sapmed.ac.jp

ORCID:

0000-0002-7949-1628 (Yuki Saito)

0000-0003-2832-3656 (Takako S. Chikenji)

Supplementary Figures

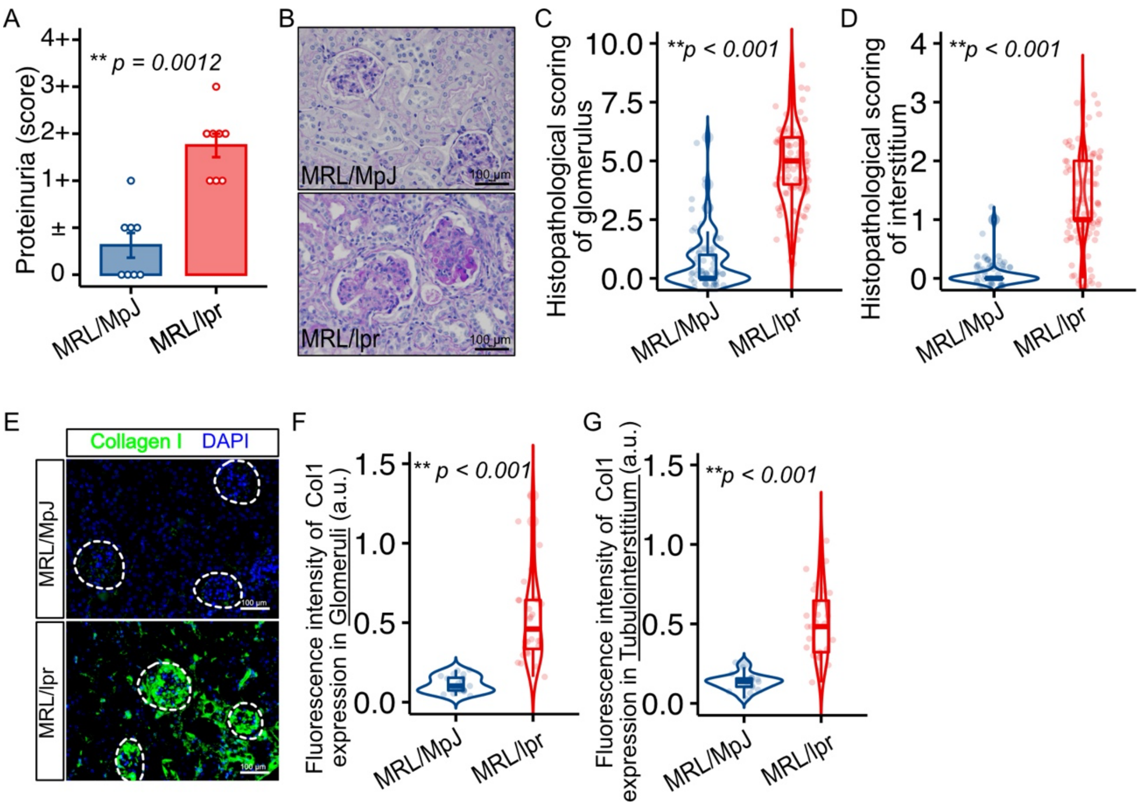

Supplemental Figure 1. Histopathology and collagen I expression in the kidneys of MRL/lpr mice. (A) Semi-quantitative data of proteinuria in MRL/lpr and MRL/MpJ mice. Data are presented as means±SEM. p-values were determined by the two-tailed Student's t-test. (B) Representative images of periodic acid-Schiff staining in kidney sections from MRL/lpr and MRL/MpJ mice. Scale bars represent 100 µm. (C–D) Quantification of histopathological scoring of glomeruli and tubulointerstitium. Data are presented as violin plots, dot plots, and box-and-whisker plots. The p-values were determined using the two-tailed Student's t-test. ***P*<0.01, ****P*<0.001 for MRL/lpr vs control (MRL/MpJ). (E) Representative images of immunohistochemical staining for type I collagen (green) and nuclei (blue) in kidney sections from MRL/lpr and MRL/MpJ mice. Scale bars represent 100 µm. (F–G) Quantification of type I collagen expression in glomeruli and tubulointerstitium by fluorescence intensity (arbitrary units, a.u.). Data are presented as violin plots, dot plots, and box-and-whisker plots. *P*-values were determined using the two-tailed Student's t-test. ***P*<0.01, ****P*<0.001 for MRL/lpr vs. MRL/MpJ (control).

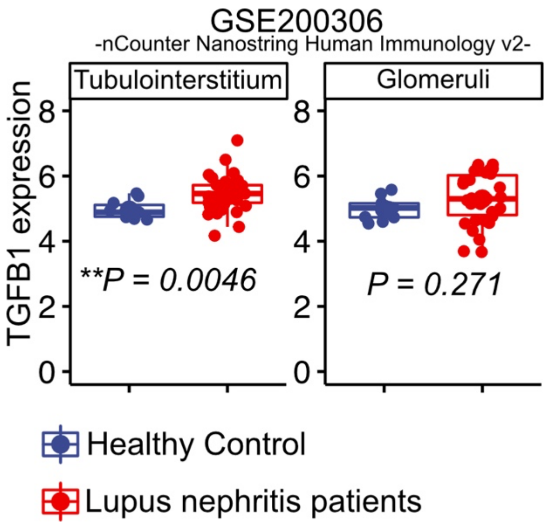

Supplemental Figure 2. TGFB1 gene expression was increased in the tubulointerstitium but not in glomeruli in lupus nephritis patients. Data are presented as medians with IQRs and 1.5 times the IQR, and are displayed as dot plots and box-and-whisker plots. *P*-values were determined using the two-tailed Student's t-test.

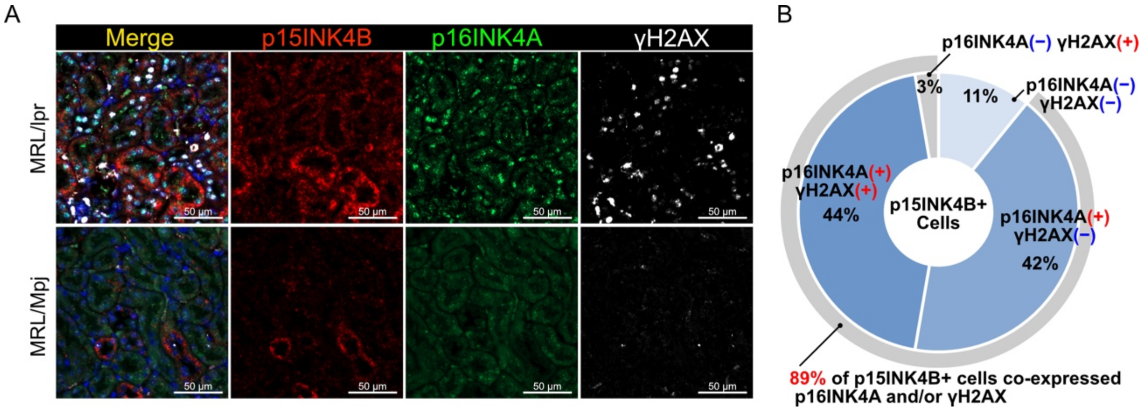

Supplemental Figure 3. p15^{INK4B}, p16^{INK4A}, and γH2AX expression in the kidneys of MRL/lpr and MRL/MpJ mice. (A) Representative images showing kidney tissue following immunostaining for p15^{INK4B}, p16^{INK4A}, and γH2AX. Tissue samples were collected from the kidneys of MRL/lpr and MRL/MpJ mice. (B) Pie chart showing proportions of cells positive for any or all of p15^{INK4B}, p16^{INK4A}, and γH2AX.

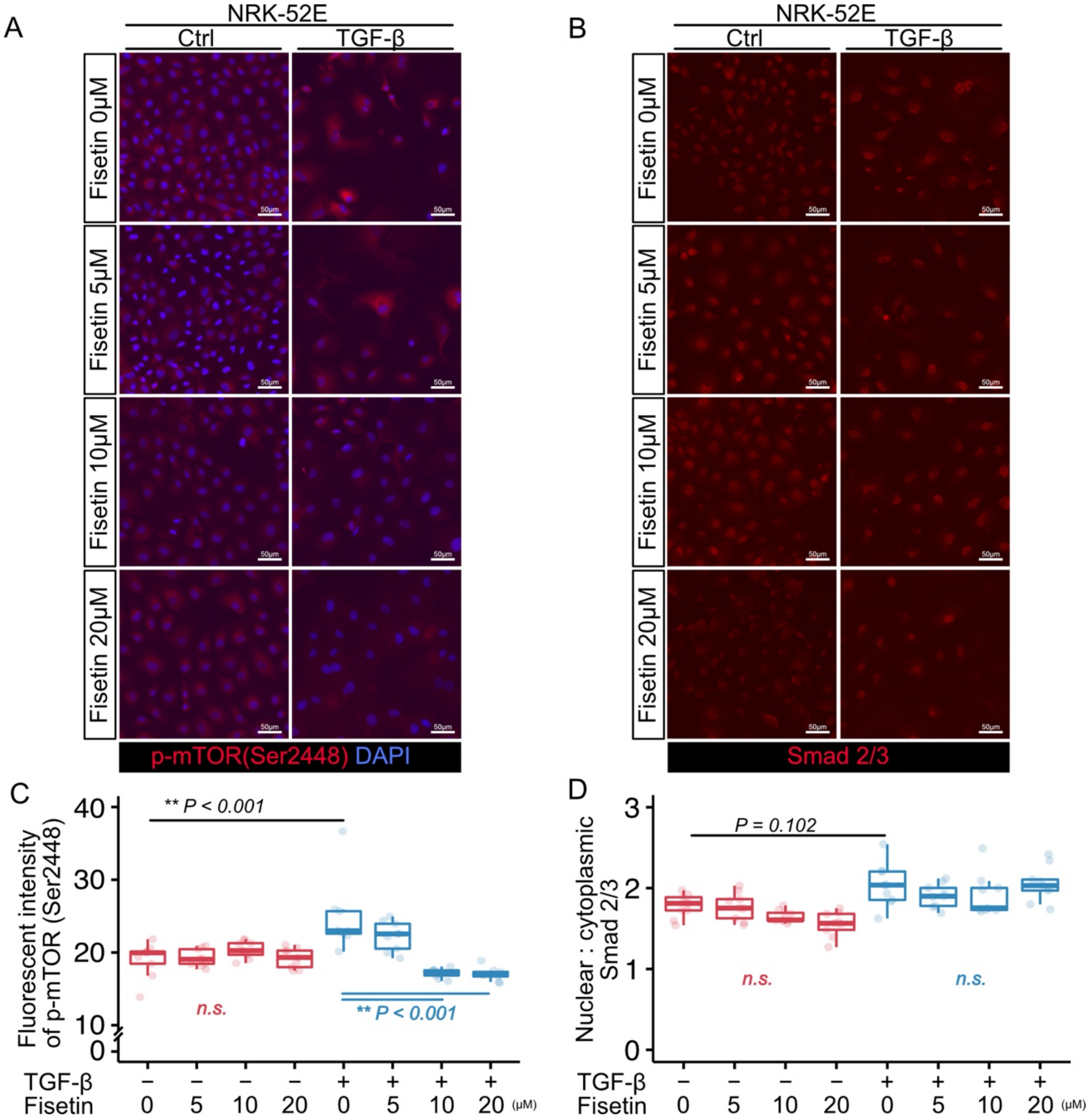

Supplemental Figure 4. Fisetin decreased phospho-mTOR in a Smad2/3-independent manner in TGF-β1–treated senescent renal tubular cells. (A and B) Representative images of phospho-mTOR and Smad2/3 staining in TGF-β1–treated senescent NRK-52E cells with or without fisetin treatment (0, 5, 10, 20 μM). (C and D) Fluorescence intensities were used to quantify phospho-mTOR and Smad2/3 expression levels in NRK-52E cells. *P*-values were determined using two-way ANOVA adjusted by Tukey's method (**P<0.01).

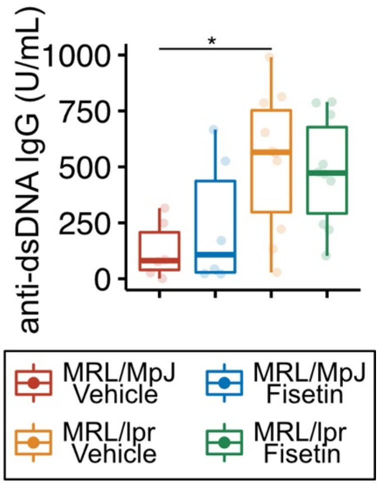

Supplemental Figure 5. Fisetin improved serum dsDNA autoantibody concentrations in MRL/lpr mice. Data are presented as medians with IQRs and 1.5 times the IQR, and are displayed as dot plots and box-and-whisker plots. *P*-values were determined using one-way ANOVA adjusted by Tukey's method (*$P<0.05$).

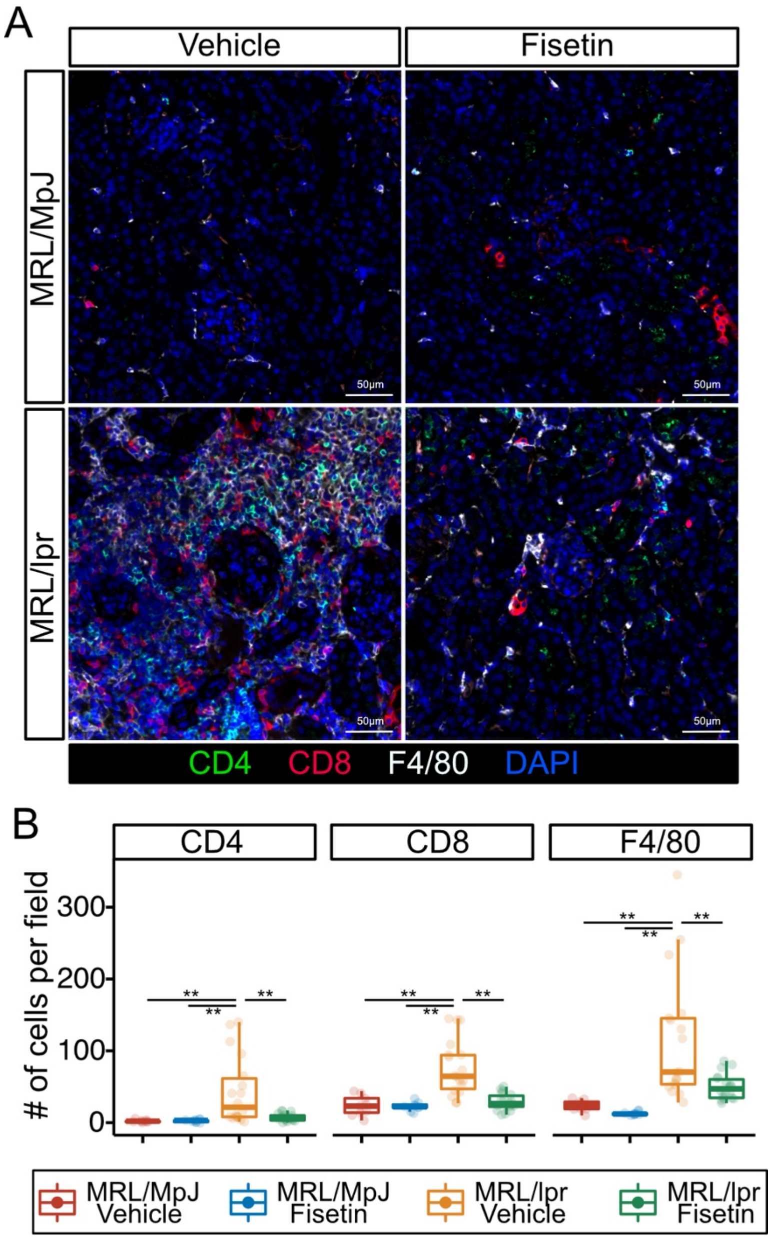

Supplemental Figure 6. Fisetin decreased the numbers of CD4-, CD8-, and F4/80-positive cells in the kidneys of MRL/lpr mice. (A) Representative images of kidney tissue following immunostaining for CD4, CD8, and F4/80. (B) Quantification of the numbers of CD4-, CD8-, and F4/80-positive cells in the tubulointerstitium. Data are presented as dot plots and box-and-whisker plots. *P*-values were determined using one-way ANOVA adjusted by Tukey's method (***P*<0.01).

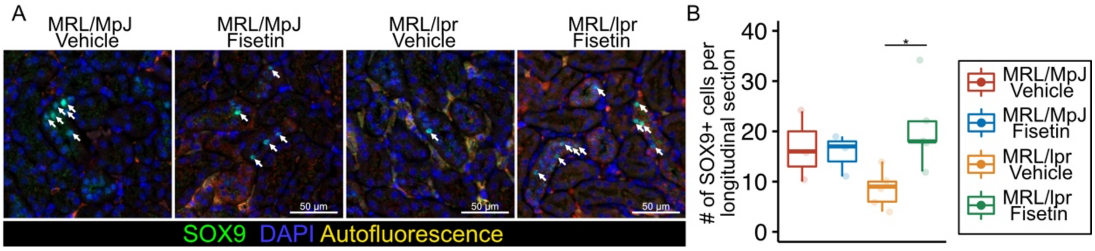

Supplemental Figure 7. Fisetin increased the number of Sox9-positive cells in MRL/lpr mice. (A) Representative images of kidney tissue following immunostaining for Sox9 (green), DAPI (blue), Autofluorescence (yellow). (B) Quantification of the number of Sox9-positive cells in tubules. Data are presented as dot plots and box-and-whisker plots. *P*-values were determined using one-way ANOVA adjusted by Tukey's method (*$P<0.05$).

Supplementary Table 1.specific primer sequence

Mouse specific primer sequence used for real-time PCR

Gene	*Forward*	*Reverse*
Acta2	5' ACGCTGAAGTATCCGA 3'	5' CATTTTCTCCCGGTTGG 3'
Cdkan2a(P19)	5' GGGTCGCAGGTTCTTGGTC 3'	5' AATCTGCACCGTAGTTGAGCA 3'
Cdkn1a(p21)	5' TCCCGACTCTTGACATTGCT 3'	5' TGCAGAAGGGGAAGTATGGG 3'
Cdkn2a(P16)	5' GGGTTTCGCCCAACGCCCCGA 3'	5' TGCAGCACCACCAGCGTGTCC 3'
Cdkn2b	5' AATAACTTCCTACGCATTTTCTGC 3'	5' CCCTTGGCTTCAAGGTGAG 3'
Col1a1	5' CCTCAGGGTATTGCTGGACAAC 3'	5' CAGAAGGACCTTGTTTGCCAGG 3'
Col3a1	5' GACCAAAAGGTGATGCTGGACAG 3'	5' CAAGACCTCGTGCTCCAGTTAG 3'
Fn1	5' ATCTGGACCCCTCCTGATAGT 3'	5' GCCCAGTGATTTCAGCAAAGG 3'
Il1b	5' TGCCACCTTTTGACAGTGATG 3'	5' TGATGTGCTGCTGCGAGATT 3'
Il1b	5' TGGACCTTCCAGGATGAGGACA 3'	5' GTTCATCTCGGAGCCTGTAGTG 3'
Il6	5' TGATTGTATGAACAACGATGATGC 3'	5' GGACTCTGGCTTTGTCTTTCTTGT 3'
Mki67	5' CCTTTGCTGTCCCCGAAGA 3'	5' GGCTTCTCATCTGTTGCTTCCT 3'
Nfkb1	5' GCTGCCAAAGAAGGACACGACA 3'	5' GGCAGGCTATTGCTCATCACAG 3'
Pdgfra	5' GCAGTTGCCTTACGACTCCAGA 3'	5' GGTTTGAGCATCTTCACAGCCAC 3'
Tgfb1	5' GCCTGAGTGGCTGTCTTTTGA 3'	5' CACAAGAGCAGTGAGCGCTGAA 3'
Tnf	5' GGTGCCTATGTCTCAGCCTCTT 3'	5' GCCATAGAACTGATGAGAGGGAG 3'
Vim	5' CGGAAAGTGGAATCCTTGCAGG 3'	5' AGCAGTGAGGTCAGGCTTGGAA 3'
Actb	5' CATTGCTGACAGGATGCAGAAGG 3'	5' GCCATAGAACTGATGAGAGGGAG 3'
Gapdh	5' AGGTCGGTGTGAACGGATTTG 3'	5' AGCAGTGAGGTCAGGCTTGGAA 3'

Rat specific primer sequence used for real-time PCR

Gene	*Forward*	*Reverse*
Cdkn2b	5' ATGTGAGGCACTGAAGTGGG 3'	5' CCTCAAGGGAACTGGGTCAC 3'
Gapdh	5' CTCTCTGCTCCTCCCTGTTC 3'	5' TACGGCCAAATCCGTTCACA 3'
Actb	5' GTCCACCCGCGAGTACAAC 3'	5' GGATGCCTCTCTTGCTCTGG 3'
Hprt1	5' GTCATGTCGACCCTCAGTCC 3'	5' TTGGGGCTGTACTGCTTGAC 3'
Gusb	5' GGGTGTGGTATGAACGGGAA 3'	5' CTCTGGACCAGCTTGGTGAT 3'
